# Acid Hydrolysis of Wheat Gluten Induces Formation of New Epitopes but Does Not Enhance Sensitizing Capacity by the Oral Route: A Study in “Gluten Free” Brown Norway Rats

**DOI:** 10.1371/journal.pone.0107137

**Published:** 2014-09-10

**Authors:** Stine Kroghsbo, Nanna B. Andersen, Tina F. Rasmussen, Susanne Jacobsen, Charlotte B. Madsen

**Affiliations:** 1 National Food Institute, Technical University of Denmark, Søborg, Denmark; 2 Enzyme and Protein Chemistry, Department of Systems Biology, Technical University of Denmark, Lyngby, Denmark; New York State Dept. Health, United States of America

## Abstract

**Background:**

Acid hydrolyzed wheat proteins (HWPs) are used in the food and cosmetic industry as emulsifiers. Cases of severe food allergic reactions caused by HWPs have been reported. Recent data suggest that these reactions are caused by HWPs produced by acid hydrolysis.

**Objectives:**

To examine the sensitizing capacity of gluten proteins *per se* when altered by acid or enzymatic hydrolysis relative to unmodified gluten in rats naïve to gluten.

**Methods:**

High IgE-responder Brown Norway (BN) rats bred on a gluten-free diet were sensitized without the use of adjuvant to three different gluten products (unmodified, acid hydrolyzed and enzymatic hydrolyzed). Rats were sensitized by intraperitoneal (i.p.) immunization three times with 200 µg gluten protein/rat or by oral dosing for 35 days with 0.2, 2 or 20 mg gluten protein/rat/day. Sera were analyzed for specific IgG and IgE and IgG-binding capacity by ELISA. IgE functionality was measured by rat basophilic leukemia (RBL) assay.

**Results:**

Regardless of the route of dosing, all products had sensitizing capacity. When sensitized i.p., all three gluten products induced a strong IgG1 response in all animals. Acid hydrolyzed gluten induced the highest level of specific IgE but with a low functionality. Orally all three gluten products induced specific IgG1 and IgE but with different dose-response relations. Sensitizing rats i.p. or orally with unmodified or enzymatic hydrolyzed gluten induced specific IgG1 responses with similar binding capacity which was different from that of acid hydrolyzed gluten indicating that acid hydrolysis of gluten proteins induces formation of ‘new’ epitopes.

**Conclusions:**

In rats not tolerant to gluten acid hydrolysis of gluten enhances the sensitizing capacity by the i.p. but not by the oral route. In addition, acid hydrolysis induces formation of new epitopes. This is in contrast to the enzymatic hydrolyzed gluten having an epitope pattern similar to unmodified gluten.

## Introduction

Gluten proteins are wheat storage proteins, constituting about 10% of wheat, and give wheat dough functional properties such as water absorption capacity, viscosity and elasticity, which contribute to the unique baking properties [1]–[3]. Gluten proteins are among the most complex proteins in nature containing hundreds of components present as monomers, oligomers and polymers. Oligomers and polymers are linked by disul­phide bonds between the S-containing amino acid cysteine, which make up approximately 2% of gluten. Based on their physical characteristics, gluten proteins can be divided into gliadins and glutenins. Gliadins, also known as wheat prolamins, are soluble in alcohol and are mainly present in gluten as monomers interacting by non-covalent forces. Glutenins are soluble in dilute acid and are mainly present as aggregated proteins linked by intra- and interchain disulphide bonds. Gliadins and glutenins consist mainly of glutamine, proline and the essential amino acid phenylalanine [3], [4]. Another unique feature about gluten proteins is their structures, which are dynamic, comprise interchanging conformations and do not unfold when exposed to heating like most other proteins [5].

Gluten proteins are by definition not soluble in water but enzymatic or acid hydrolysis can alter their structure and size and thereby resulting in soluble protein hydrolysates. Acid hydrolyses also result in partial deamidation of the proteins [6]. Acid hydrolyzed wheat gluten obtain emulsifying properties which make them useful in food products such as coffee creamers and shake-and-bake products but also in cosmetic products for hair and body [6], [7].

The digestibility of wheat depends on the degree of processing and grinding [8]. Results from *in* *vitro* digestion studies of wheat proteins indicate that gluten proteins partially resist digestion in the stomach and intestine and thus may be absorbed as structurally intact proteins [9]. However, when gluten proteins are deamidated, it may result in increased digestibility [10]. In general, hydrolysis can alter the way proteins are broken down, digested and absorbed across the gut mucosal barrier [5]. Hence, there is a possibility that the mucosal immune system is not exposed to hydrolyzed gluten proteins in the same way as gluten proteins [5], [11].

The default immunological reaction to ingested food proteins is tolerance. However, in susceptible individuals an otherwise harmless food or food component can induce an IgE-mediated food allergy, which involves an abnormal response of the immune system to specific food proteins. IgE-mediated wheat allergy appears in three different types; wheat food allergy, wheat-dependent exercise-induced anaphylaxis (WDEIA) and baker’s asthma, which must be distinguished from each other [12]. Recently allergy to HWPs was recognized as a fourth type of IgE-mediated allergy to wheat. Most interestingly, HWPs have been reported to cause food allergic reactions in patients who are tolerant to wheat [11], [13]–[17]. Allergic skin reactions after application of cosmetic products containing HWPs have also been described [7], [14], [15], [18], [19]. However, the type of hydrolysis (enzymatic or acid) in the papers on HWP’s is not always clearly defined. In Japan the use of a facial soap containing acid hydrolyzed wheat protein has caused allergic skin symptoms and WDEIA i.e. allergic reactions to wheat products [20], [21]. A wide spectrum of wheat proteins belonging to the albumin/globulin fraction (salt-soluble), gliadin fraction (alcohol-soluble) and glutenin fraction (soluble in dilute acid) [9], [11], [13]–[15], [19], [22], [23] have been reported to be involved in IgE-mediated allergic reactions to wheat. However, at present no study has clearly demonstrated a correlation between the type of IgE-mediated wheat allergy and pattern of IgE-reactivity to a specific fraction of wheat proteins. In general, food allergens have a high resistance to heat. This is also the case of gluten proteins that contain potentially thermo-stable IgE-epitopes [5], [9], [25]. The route of sensitization can occur via the gastrointestinal tract, via the respiratory tract (wheat flour) or via the skin [12], [25]–[27].

Solubility increases when gluten is hydrolyzed by enzymes. This type of product is used in food as a protein supplement. When gluten is hydrolyzed by acid, emulsifying properties are created. This type of product is used as emulsifier in food and in cosmetics.

To investigate if hydrolysis alters the immunogenicity of gluten proteins we have studied the sensitization capacity of unmodified, enzymatic hydrolyzed and acid hydrolyzed wheat gluten. To be able to compare the immune response *per se* the influence from gluten tolerance was excluded by performing the studies in Brown Norway rats naïve to gluten (bred for more than three generations on a gluten free diet). Rats were dosed intraperitoneally or orally.

## Material and Methods

### Ethics statement

Animal experiments were carried out at the DTU Food (Mørkhøj, Denmark) facilities. Ethical approval was given by the Danish Animal Experiments Inspectorate. The authorization number given: 2009/561-01710. The experiments were overseen by the National Food Institutes in-house Animal Welfare Committee for animal care and use.

### Wheat gluten products

Wheat gluten (unmodified), enzymatic hydrolyzed wheat gluten and acid hydrolyzed wheat gluten were provided by Tereos Syral, Aalst, Belgium. The enzyme used in the production of enzyme hydrolyzed gluten was an endoprotease with no transglutaminase activity. Compared to the conditions of acid hydrolysis, the enzymatic reaction occurs at more neutral pH and moderate temperature. The acid hydrolyzed gluten is produced at very low pH (below pH 2.5) and at high temperature (above 80°C). For technical information on gluten products see Table 1. It can be seen that acid hydrolysis results in substantial deamidation. The level of endotoxin in gluten proteins were analyzed according to manufacturer’s instructions (Pierce LAL Chromogenic Endotoxin Quantitation kit; Fisher Scientific ApS, Slangerup, Denmark). Endotoxin levels were below 50 EU/mg gluten protein for all products.

**Table 1:** Technical information on unmodified, enzymatic hydrolyzed and acid hydrolyzed wheat gluten provided by Tereos Syral.

	Unmodifiedwheat gluten	Enzymatichydrolyzed wheatgluten	Acid hydrolyzed wheatgluten
**% relative protein (SDS soluble)** *			
F1>779.6 kDa	9.8	0.7	0.9
779.6 kDa>F2>96.7 kDa	26.0	4.3	13.6
96.7 kDa>F3>58.1 kDa	7.7	2.8	9.5
58.1 kDa>F4>20.5 kDa	42.7	21.7	40.3
20.5 kDa>F5>6.2 kDa	12.7	60.4	34.4
6.2 kDa>F6>0.47 kDa	1.1	10.2	1.3
**Degree of deamidation (%)** ¤	∼3	∼3	60
**Emulsifying capacity** †	-	-	950
**Solubility in PBS (%)** ‡	35	100	98

*Weight distribution for SDS soluble protein in gluten products expressed as relative % of different fractions. 15.3% of unmodified gluten was not soluble in SDS whereas enzymatic and acid hydrolyzed gluten products were completely soluble.

¤ Degree of deamidation was measured by a titrimetric determination of the free ammonia in native, enzyme hydrolyzed and acid hydrolyzed wheat gluten, expressed as % of the free ammonia after total deamidation of wheat gluten.

† Emulsifying capacity denotes the maximum amount of oil that is emulsified by a standard amount of protein (g oil/g product).

‡ Solubility of gluten proteins in phosphate buffered saline (PBS) based on protein concentration measured by amino acid analysis. Determined as % protein extracted from 1 mg/mL solutions of gluten products. Own results.

-: not tested.

### Preparation of soluble fractions of wheat gluten products for analysis of antibody response

Gluten products were dissolved by carefully adding 1 mg/mL unmodified gluten or hydrolyzed gluten products to phosphate buffered saline (PBS; 137 mM NaCl, 3 mM KCl, 8 mM Na_2_HPO_4_, 1 mM KH_2_PO_4_; pH 7.2) before magnetic stirring for 2 hours at room temperature (RT) followed by ultrasonication for 3–5 hours. After centrifugation at 2000×*g* for 20 min. at 4°C, supernatants were collected and stored at ÷20°C. Protein concentrations in supernatants were determined by amino acid analysis [28].

As approximately 35% of proteins in unmodified gluten were soluble in PBS, gluten proteins were extracted by ethanol (gliadin fraction) followed by sodium oleate (glutenin fraction) for measurement of the specific antibody response to ‘total’ gluten proteins. Briefly, unmodified gluten were suspended in 50% ethanol (2 mg gluten protein/mL), mixed carefully for 10 min. before ultrasonication for 2–3 hours. The gliadin fraction was obtained by collecting the supernatant after centrifugation (2000×*g*, RT, 5 min.). The glutenin fraction was obtained by suspending the remaining pellet in 0.1% (w/v) sodium oleate solution before carefully mixing for 10 min. followed by ultrasonication for 2 hours and collection of supernatant after centrifugation (2000×*g*, RT, 15 min.). Gliadin and glutenin fractions were stored at RT.

### Animals

Inbred high IgE-responder Brown Norway (BN) rats were from our in-house colony at the National Food Institute (DTU, Denmark). Rats were bred and kept for at least three generations on a gluten free diet developed and produced at the National Food Institute (DTU, Denmark) [29] to ensure immunologically naïve animals with respect to wheat proteins. The diet was analyzed for wheat proteins using a sensitive commercial kit for detection of gliadin peptide fragments in accordance with manufacturer’s instructions (Art.no. R7011, RIDASCREEN Gliadin competitive; Food Diagnostics, Grenaa, Denmark). For sensitization studies, 4–6 weeks old rats, were allocated into groups and housed in macrolon cages (two/cage) at 22±1°C, relative humidity 55±5%, air change 10 times/h, and electric light from 9.00 am – 9.00 pm. Diet and acidified water (pH 3.5) were given *ad libitum*. Animals were inspected twice daily and body weights recorded weekly. At termination of sensitization experiments all animals were anaesthetized by hypnorm-dormicum and killed by exsanguinations. Animal experiments were carried out at the National Food Institute (DTU, Denmark) facilities and performed under conditions approved by the Danish Animal Experiments Inspectorate and by the in-house Animal Welfare Committee for animal care and use.

### Animal sensitization studies

Sensitization capacity of unmodified gluten and hydrolyzed gluten products were studied in two different rat models; an i.p. model based on intraperitoneal immunization with the soluble fraction of gluten products and an oral model based on intragastric dosing of rats with ‘whole’ gluten products suspended in water.

For each of the gluten products, positive control sera were produced in BN rats by i.p. immunization with 200 µg gluten product adsorbed on Alhydrogel (Brenntag Biosector, Frederikssund, Denmark) at day 0 and with 20 µg gluten product suspended in 0.9% NaCl at day 14, 21 and 28. Rats were immunized with 0.2 mL per rat and blood was collected at sacrifice (day 35).

Sera from all animal experiments were stored at ÷20°C until analysis.

#### I.p. study with gluten products

Groups of 16 BN rats (8 rats per sex) were immunized i.p. on day 0, 14 and 28 with unmodified gluten or hydrolyzed gluten products extracted in PBS (200 µg protein/rat/immunization). Blood was collected on day 35.

#### Oral sensitization studies with gluten products

Only female BN rats (12 per group) were included in these studies as it has previously been shown that significantly more responders are found amongst female BN rats than among males when dosed by gavage [30]. Rats were dosed by gavage each day for 35 days (day 1–35) with 0.2, 2 or 20 mg of unmodified gluten or hydrolyzed gluten products suspended in PBS. Suspensions were carefully mixed between dosing of rats to avoid precipitation. For all feeding studies, the dosing volume was 0.5 mL per rat per day and blood was collected on day 0, 14, 28 and 42 (sacrifice).

### Enzyme-Linked Immuno Sorbent Assay (ELISA)

Sera from sensitization studies were analyzed for specific IgG1 and IgE by ELISA. Positive and negative serum control pools were included on each plate. Antibody titers were expressed as log2 titers and calculated using four-parameter analysis, Gen5 (Bio-Tek Instruments Inc., Winooski, VT, USA). Detection limits were determined as the mean absorbance for the negative control serum+3 standard deviations (SD). For all ELISA experiments washing procedures and development of enzymatic reaction were as described previously [31]. All reagents and incubation periods were at RT for 1 hour unless otherwise noted.

#### Detection of specific IgG1 by direct ELISA

For detection of specific IgG1, plates (96-well, MaxiSorp; Nunc, Roskilde, Denmark) were coated overnight at 4°C with 100 µL/well of 2 µg/mL of the soluble fraction of unmodified gluten or hydrolyzed gluten products, gliadin fraction or glutenin fraction in carbonate buffer (15 mM Na_2_CO_3_, 35 mM NaHCO_3_; pH 9.6). Plates were incubated with serially two-fold diluted rat sera in PBS containing 0.01% Tween (PBS-T) before incubation with horseradish peroxidase (HRP)-labeled mouse anti-rat IgG1 (1∶20,000; SouthernBiotech cat. no. 3060-05, Birmingham, AL, USA) in PBS-T followed by development with TMB-one (Kem-En-Tec, Copenhagen, Denmark) for 10 min in the dark. Absorbance values for detection limits were calculated to 0.1 for all IgG1 assays.

#### Inhibition ELISAs for examination of IgG1-binding capacity

Assay procedures were as described for measurement of specific IgG1 except that sera were preincubated with inhibitor solutions. Sera from individual rats were diluted to reach an OD between 0.8 and 1.0 and preincubated for 1 hour at RT with serial ten-fold dilutions of the soluble fraction of gluten products (50 pg/mL to 50 µg/mL) before triplicates of serum/inhibitor mix (and sera with no inhibitor as a control) were added to the wells. Results were expressed as % *B*/*B_0_* where *B* corresponds to the specific IgG1-binding to immobilized gluten protein when a known concentration of inhibitor is present and *B_0_* corresponds to the binding in the absence of inhibitor. For each serum pool, the concentration of inhibitor that inhibits 50% of the binding to the immobilized antigen (IC_50_) was determined, where an increase in IC_50_ value is correlated to a lower IgG1-reactivity of the product used as inhibitor. Inhibition curves were analyzed by GraphPad Prism (GraphPad Software, San Diego, CA, USA) to examine if curves were parallel (no significant difference between slopes) which is important for appropriate comparison of IC_50_ values.

#### Detection of specific IgE by antibody-capture ELISAs

To avoid the interference of the much higher level of IgG than IgE in rat sera, assays based on selective IgE capture were established for detection of specific IgE responses. Plates were coated overnight at 4°C with 0.5 µg/mL of mouse anti-rat IgE (HPMAB-123 HybriDomus, Cytotech, Hellebæk, Denmark) in carbonate buffer. After blocking of remaining active sites for 1 hour at 37°C with PBS-T containing 3% bovine serum albumin (BSA; A2153, Sigma, Copenhagen, Denmark), plates were incubated with serially two-fold diluted rat sera and then with biotin-coupled gluten products or gliadin diluted in PBS-T containing 3% BSA (biotin-gluten 1∶200, biotin-enzymatic hydrolyzed gluten 1∶1500, biotin-acid hydrolyzed gluten 1∶800 and biotin-gliadin 1∶250). After washing, plates were incubated with HRP-labeled NeutrAvidin (cat. no. 31030; ThermoScientific, Slangerup, Denmark) diluted in PBS-T containing 3% BSA (diluted 1∶4000 for detection of anti-gluten IgE, anti-acid hydrolyzed gluten IgE and anti-gliadin IgE or diluted 1∶8000 for detection of anti-enzymatic hydrolyzed gluten IgE). Plates were developed for 10 min in the dark and absorbance values for detection limits were calculated to 0.1 for all IgE assays.

Biotin was coupled to each of the gluten products or gliadin using a biotin protein-labeling kit (Pierce EZ-Link Sulfo-NHS-LC-Biotinylation Kit; ThermoScientific) in accordance with the manufacturer’s instructions. For gluten products, proteins soluble in PBS were used for biotinylation whereas the gliadin fraction of unmodified gluten was dissolved in 50% ethanol before biotinylation. The highest stability of biotin-coupled gluten proteins was obtained when biotin-gliadin was stored at ÷20°C and biotinylated gluten products were stored at 4°C.

### Rat Basophilic Leukemia (RBL) Assay

The biological functionality of specific IgE was examined by RBL assay as described previously [29] with minor modifications. Briefly, RBL-2H3 cells (kindly provided by Prof. Stefan Vieths, Paul-Ehrlich Institute, Langen, Germany) were harvested in the stationary phase, after over-confluence had been reached. Cells were re-suspended (1×10^6^ cells/mL) in assay medium (Eagle MEM supplemented with 100 U/100 µg/mL penicillin/streptomycin solution, 2.5 µg/mL amphotericin, and 2 mM L-glutamine) and incubated overnight (100 µL/well) in flat-bottomed cell culture microtitre plates (Nunc) for attachment. Attached cells were sensitized passively with 50 µL/well of undiluted rat serum pools for 2 hours (37°C, 5% CO_2_) and then washed twice before incubation with 100 µL/well of five-fold diluted extracts of gluten products (0.02–350 µg protein/mL) for 60 min for cross-linking. After incubation plates were centrifuged (200×*g*, 5 min) and 50 µL of supernatant (Specific release) was transferred to a microtitre plate (Nunc) for measurement of *β*-hexosaminidase enzymatic activity. Total release from remaining intact cells was measured for each well by addition of detergent (50 µL/well of 0.1% Triton X-100; X100, Sigma), incubated for 30 min before centrifugation and transfer of 50 µL supernatant (Total release) to a second microtitre plate.

For control of IgE-mediated degranulation (total biological release), serum-sensitized cells were stimulated with 1.25 µg/mL of mouse anti-rat IgE (553914, BD Pharmingen, San Diego, CA, USA). Negative controls were included on each plate to measure spontaneous release (equivalent to background reading).

For each serum sample *β*-hexosaminidase release was calculated according to the following equation:
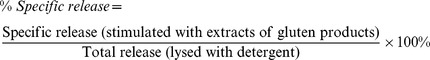



As IgE-mediated degranulation (*Total IgE release*) for serum pools were 50–60% of total release for all serum pools and no statistically significant difference was found between groups, results are expressed as percent of maximum biological release:
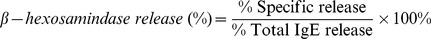



### Statistical analysis

Statistical analysis of data was carried out using GraphPad Prism version 4.00 for Windows (GraphPad Software, San Diego, CA, USA). ELISA results expressed as antibody titers or IC_50_ values were examined using non-parametric statistical analysis when distributions were not normal (Kruskal-Wallis test followed by Dunn’s multiple comparison test). One-way ANOVA followed by Tukey’s multiple comparison test was used for comparison between groups with normal distributions. Differences between experimental groups were regarded as significant when *p*≤0.05.

## Results

### Acid hydrolyzed gluten induces the highest level of specific IgE by the i.p. route

**Figure 1:**
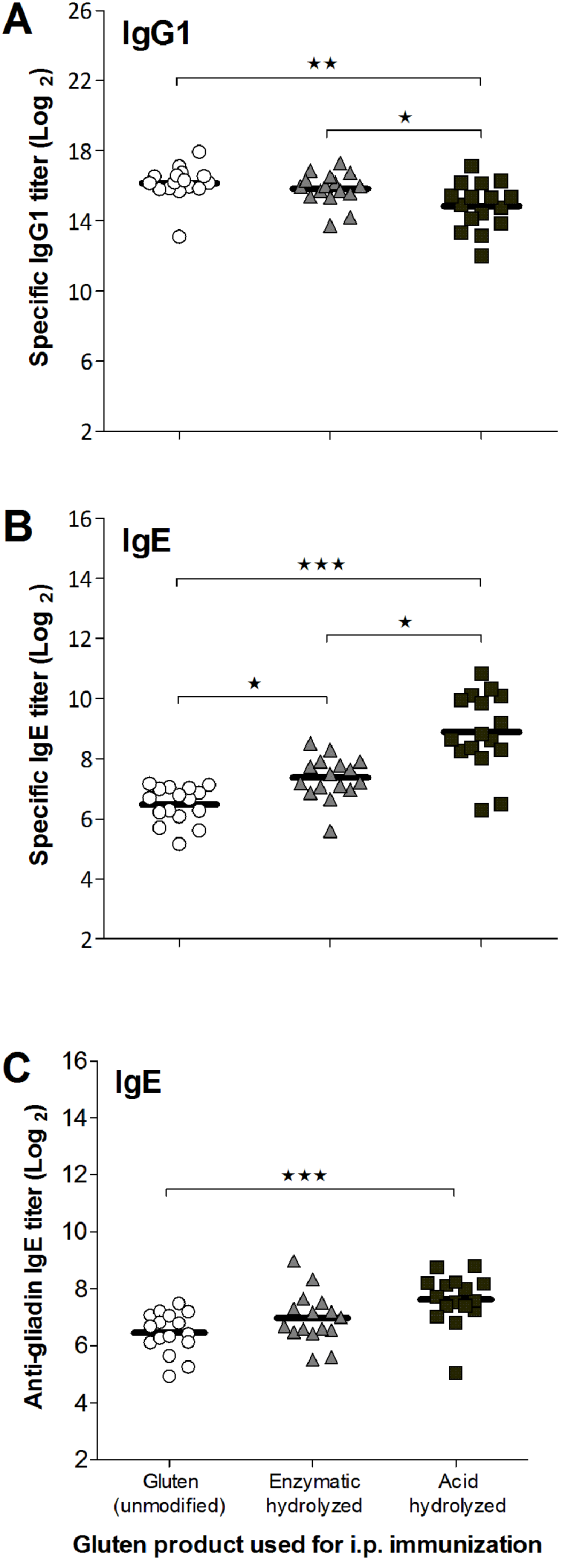
Specific IgG1 and IgE response in sera from BN rats immunized i.p. with unmodified gluten (open circles), enzymatic hydrolyzed gluten (grey triangles) or acid hydrolyzed gluten (black squares). Groups of rats were immunized i.p. on day 0, 14 and 28 with 200 µg gluten protein per rat without adjuvant. Serum samples were obtained at sacrifice (day 35) and analyzed by ELISA for specific IgG1 (*A*) and IgE (*B*) against the gluten product used for immunization and for anti-gliadin IgE (*C*). Each symbol represents an animal and horizontal bars indicate mean values. Data were analyzed by one-way ANOVA (IgG1) and non-parametrical test (IgE). Asterisks indicate statistically significant difference between groups; *: *p*≤0.05, **: *p*≤0.01, ***: *p*≤0.001.

Although unmodified gluten induced a statistically significant higher IgG1 response compared to the acid hydrolyzed gluten all three products induced a high level of specific IgG1 in all rats when immunized i.p. (Figure 1*A*). In contrast, the highest level of specific IgE was obtained by immunization with acid hydrolyzed gluten (Figure 1*B*). ELISAs for measurement of specific IgE against the different gluten products are not necessarily comparable as it is uncertain whether biotin has been equally coupled to the mixture of proteins (and hydrolysis products) present in the gluten products. However, acid hydrolyzed gluten also induced the highest level of anti-gliadin IgE (Figure 1*C*).

### Acid hydrolysis of gluten discloses or produces new epitopes

**Figure 2:**
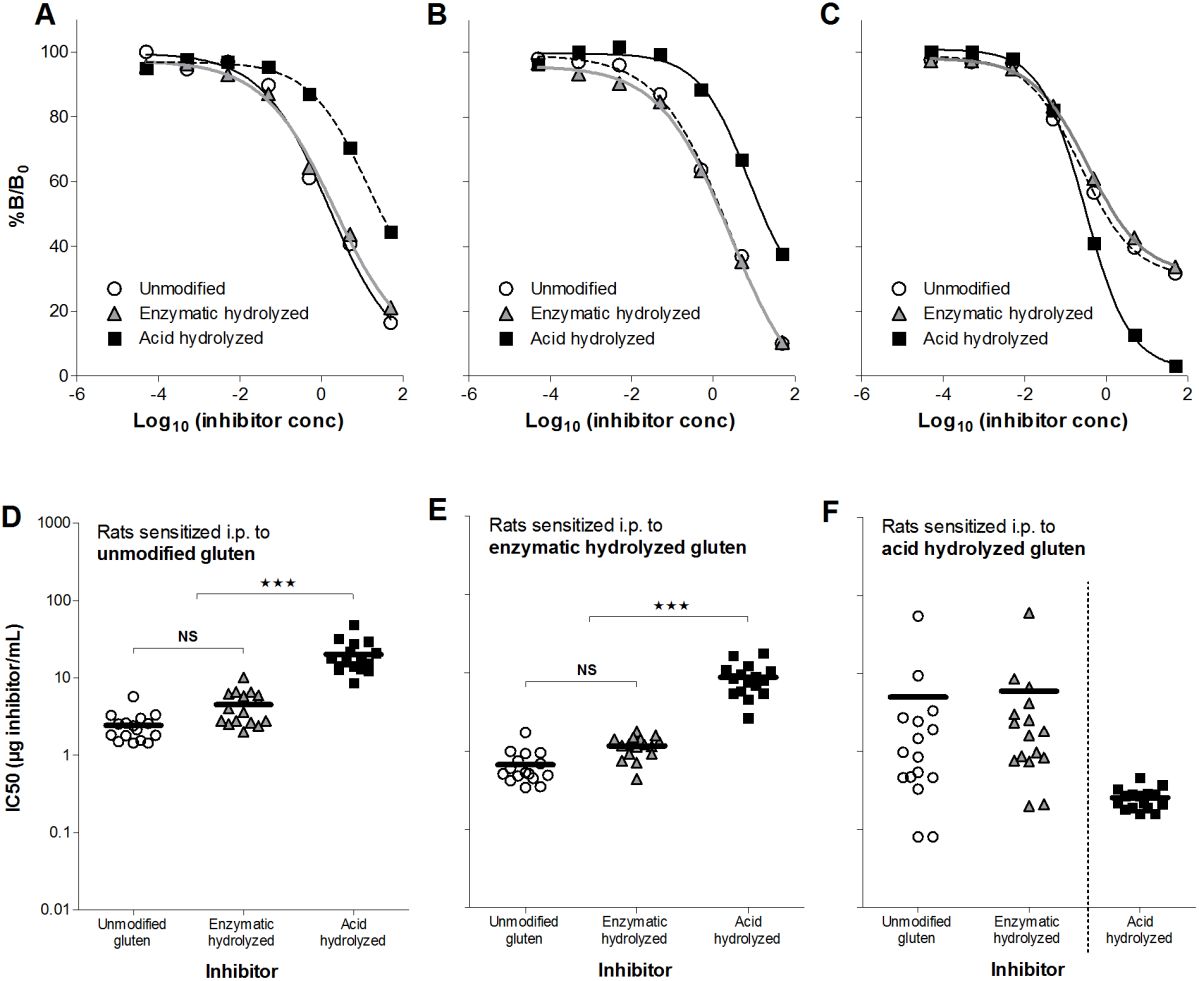
Comparison of inhibition curves and IC50 values for IgG1-binding capacity. Rats were immunized i.p. on day 0, 14 and 28 with 200 µg of unmodified gluten (*A, D*), enzymatic hydrolyzed gluten (*B, E*) or acid hydrolyzed gluten (*C, F*). Sera from individual rats obtained at sacrifice were preincubated with tenfold dilutions (50 pg/mL–50 µg/mL) of gluten products (unmodified gluten [open circles], enzymatic hydrolyzed gluten [grey triangles] or acid hydrolyzed gluten [black squares]) before determination of IgG1-binding capacity by addition to ELISA plates coated with the gluten product used for sensitization. Graphs *2A–C* show examples of inhibition curves for individual rats sensitized to unmodified (*2A*, rat no. 12), enzymatic hydrolyzed (*2B*, rat no. 31) or acid hydrolyzed (*2C*, rat no. 35) gluten. Symbols represent triplicates of the same serum/inhibitor mix and bars represent IC50 mean values for each group. Data were analyzed by one-way ANOVA. Asterisks indicate statistically significant difference between groups; ***: *p*≤0.001, NS: not significant.

Examination of IgG1-binding capacity showed that sera from rats sensitized to unmodified or enzymatic hydrolyzed gluten had equivalent binding capacity with high affinity for unmodified and enzymatic hydrolyzed gluten and significant lower affinity for acid hydrolyzed gluten (Figure 2*D, E*). The opposite was demonstrated for sera from rats sensitized to acid hydrolyzed gluten, which bound remarkably stronger to acid hydrolyzed gluten than to the other gluten products (Figure 2*F*). As shown in Figure 2, inhibition curves were identical when rats were sensitized to unmodified or enzymatic hydrolyzed gluten (2*A*, *B*). In contrast, for most rats sensitized to acid hydrolyzed gluten, the inhibition curve for acid hydrolyzed gluten was not parallel to the corresponding inhibition curves for unmodified and enzymatic hydrolyzed gluten (2*C*). Sensitization to acid hydrolyzed gluten induced IgG1 with higher avidity (mean IC50: 0.3 µg/mL) than sensitization to unmodified (mean IC50: 2.4 µg/mL) and enzymatic hydrolyzed gluten (mean IC50: 1.2 µg/mL). Together these results demonstrate that acid hydrolysis in contrast to enzymatic hydrolysis alters gluten proteins resulting in formation or disclosure of ‘new’ IgG-binding epitopes.

### The level of specific IgE does not reflect biological functionality

**Figure 3:**
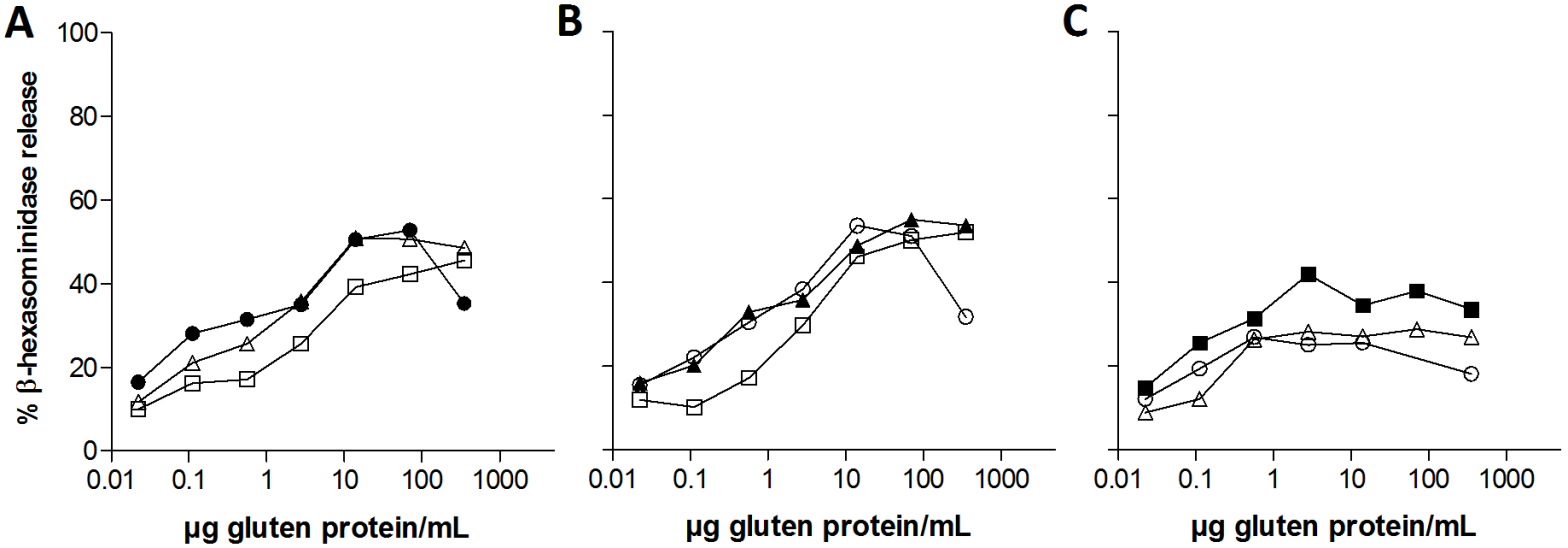
Allergen-specific degranulation of RBL cells. RBL cells were passively sensitized with undiluted serum pools of groups sensitized i.p. to unmodified gluten (*A*), enzymatic hydrolyzed gluten (*B*) or acid hydrolyzed gluten (*C*). For degranulation, cells were stimulated with five-fold dilutions of gluten products (unmodified [open circles], enzymatic hydrolyzed [grey triangles] and acid hydrolyzed [black squares] gluten, closed symbols represent the gluten product used for sensitization of rats). Data are presented as percentage allergen-specific *β*–hexosaminidase release of total release. IgE-mediated degranulation for serum pools were 50–60% for all serum pools (data not shown). Symbols represent mean values of duplicates for each serum pool.

Although i.p. sensitization to acid hydrolyzed gluten seemed to induce the highest level of specific IgE (Figure 1), a lower IgE functionality was found for sera from rats sensitized to acid hydrolyzed gluten compared to sera from rats sensitized to unmodified or enzymatic hydrolyzed gluten in terms of a lower allergen-specific degranulation of RBL cells (Figure 3). In accordance with IgG1-binding results, IgE-mediated degranulation of cells sensitized with anti-gluten or anti-enzymatic hydrolyzed gluten sera was slightly decreased when stimulated with acid hydrolyzed gluten compared to the two other products. Likewise, the highest allergen-specific degranulation of cells sensitized with anti-acid hydrolyzed gluten sera was found when stimulated with acid hydrolyzed gluten (Figure 3).

### Gluten and hydrolysis products thereof can sensitize via the oral route

**Figure 4:**
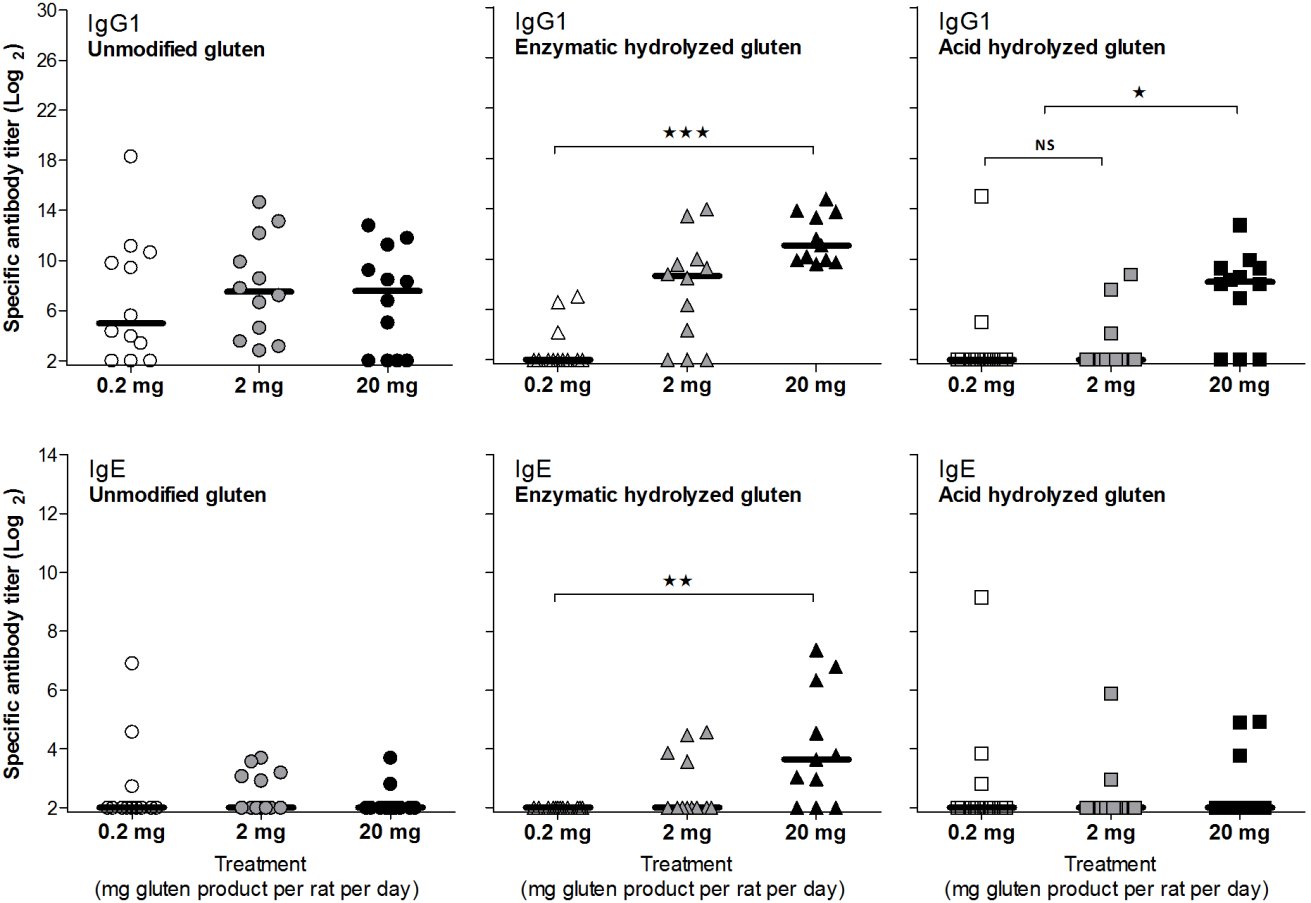
Specific antibody response in sera from BN rats dosed orally with unmodified or hydrolysis products thereof. Groups of female rats were dosed by gavage at day 1–35 with 0.2, 2 or 20 mg/rat of unmodified, enzymatic hydrolyzed or acid hydrolyzed gluten. Serum samples were obtained at sacrifice (day 42) and analyzed by ELISA for specific IgG1 and IgE. Each symbol represents an animal and horizontal bars indicate median values. Data were analyzed by non-parametrical test. Asterisks indicate statistically significant difference between groups; *: *p*≤0.05, **: *p*≤0.01, ***: *p*≤0.001, NS: not significant.

**Figure 5:**
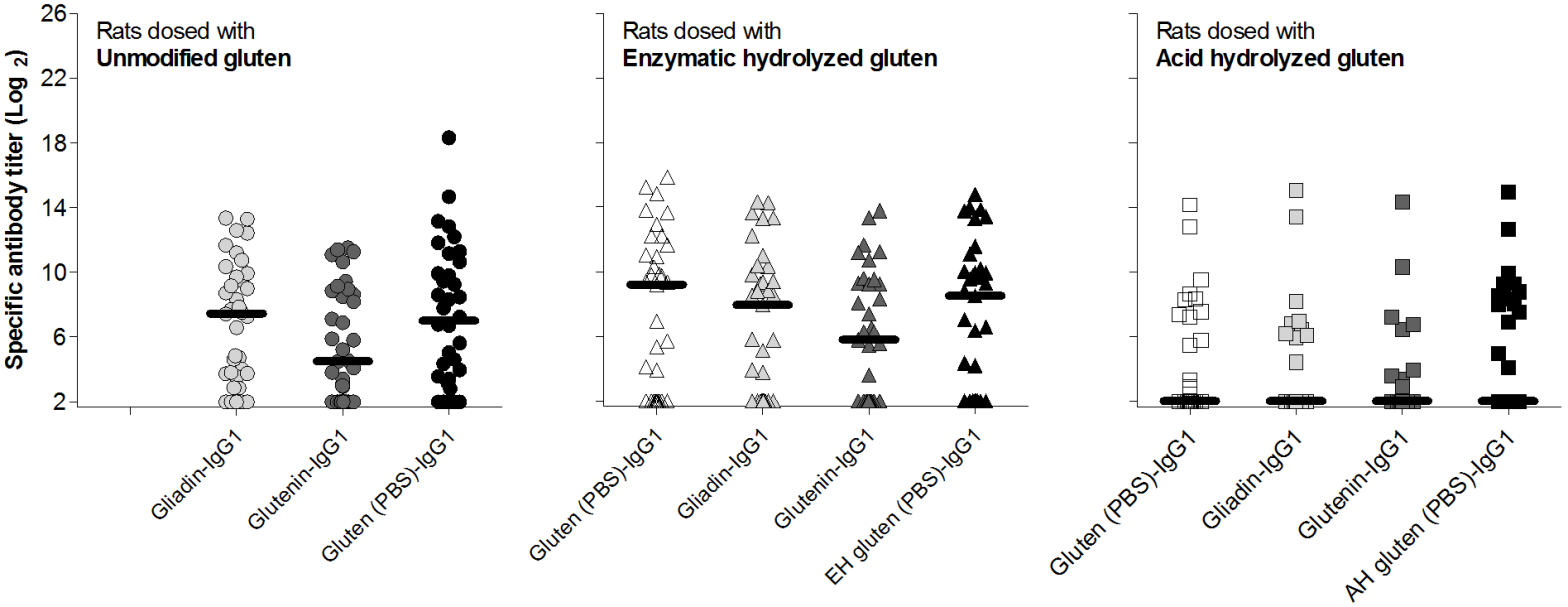
Cross-reactivity of specific IgG1 in BN rats sensitized orally to gluten products. Groups of female rats were dosed by gavage at day 1–35 with 0.2, 2 or 20 mg/rat of unmodified gluten or hydrolysis products thereof. Serum samples were obtained at sacrifice (day 42) and analyzed by direct ELISAs for specific IgG1 against unmodified gluten extracted in PBS (white symbols), gliadin fraction (light grey symbols), glutenin fraction (dark grey symbols) and the PBS extracted gluten product rats were sensitized to (black symbols). Each symbol represents an animal and horizontal bars indicate median values for groups (rats dosed with different doses of the same gluten product are shown as one group). Gluten (PBS): unmodified gluten proteins extracted by PBS, EH gluten (PBS): enzymatic hydrolyzed gluten proteins extracted by PBS, AH gluten (PBS): acid hydrolyzed gluten proteins extracted by PBS.

Sensitization capacity of unmodified gluten and hydrolysis products by the oral route was examined by dosing BN rats by gavage for 35 days with ‘whole’ gluten products suspended in PBS. A dose-response study was performed for each of the three gluten products by dosing rats with 0.2, 2 or 20 mg of gluten per rat per day for 35 days. The highest dose corresponds to 0.2% of the daily rat diet (10 gram feed/rat/day). In man eating 1.5 kg food/day, 0.2% corresponds to 3 gram of gluten (30 gram of wheat flour) or gluten product corresponding to 60 gram or 1–2 slices of bread. As shown in Figure 4, a significant IgG1 response were induced for all gluten products in the high dose groups (20 mg/rat/day) whereas at lower doses unmodified and enzymatic hydrolyzed gluten induced higher levels of specific IgG1 compared to acid hydrolyzed gluten. Levels of specific IgG1 and number of responders were clearly dose-related for enzymatic hydrolyzed gluten while unmodified gluten induced comparable IgG1 levels at all tested doses. Acid hydrolyzed gluten only induced a notable IgG1 at the highest dose. Analysis of sera from day 0, 14, 28 and 42 showed that the magnitude of specific IgG1 and number of responders increased over time for all of the tested gluten products (data not shown). Like the IgG1 response, a dose-related IgE response was only found for enzymatic hydrolyzed gluten. Only enzymatic hydrolyzed gluten induced a significant IgE response. This was at the high dose. A few rats sensitized with low dose unmodified or acid hydrolyzed gluten had remarkably high IgE titers (Figure 4). Direct IgG1 ELISAs for detection of specific IgG1 against the gluten products were based on coating with proteins, which were soluble in PBS (Table 1). To measure specific IgG1 against ‘all’ gluten proteins, the gliadin and glutenin fractions obtained from unmodified gluten were used for coating in IgG1 ELISAs. The results showed that although only a minor fraction of unmodified gluten is salt soluble the anti-gliadin IgG1 response is comparable to the IgG1 response to unmodified gluten in PBS whereas the response to glutenin is slightly lower. Identical results were found for enzymatic and acid hydrolyzed gluten (Figure 5).

### IgG1-binding after oral sensitization to acid hydrolyzed gluten differs from sensitization to unmodified or enzymatic hydrolyzed gluten

**Figure 6:**
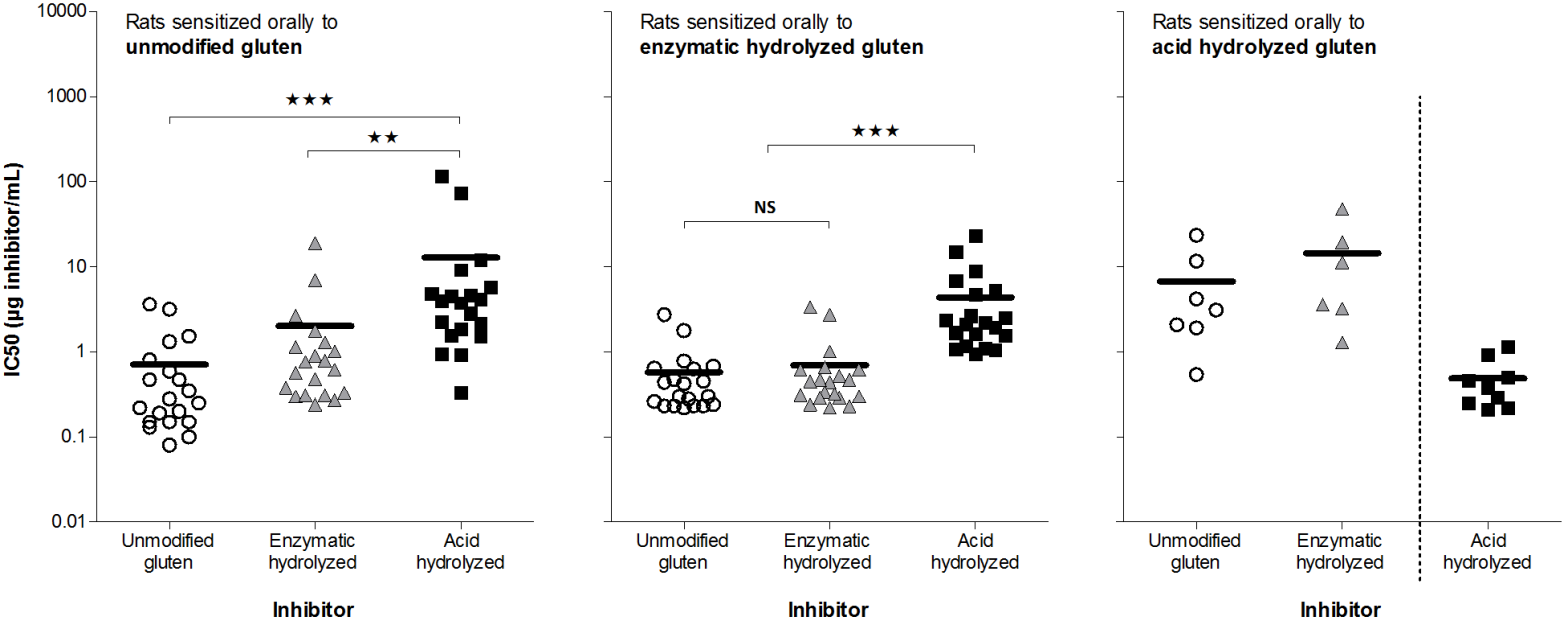
Comparison of IgG1-binding capacity of sera from oral sensitization studies with gluten products. Groups of rats were dosed by gavage at day 1–35 with 0.2, 2 and 20 mg/rat of unmodified gluten or hydrolysis products thereof. Sera from individual rats obtained at sacrifice were preincubated with tenfold dilutions of (50 pg/mL–50 µg/mL) of unmodified gluten (open circles), enzymatic hydrolyzed gluten (grey triangles) or acid hydrolyzed gluten (black squares) before measurement of IgG1-binding capacity by addition to ELISA plates coated with the gluten product used for sensitization. Only sera with an IgG1 titer >7 were analyzed by inhibition ELISA. Symbols represent triplicates of the same serum/inhibitor mix and bars represent IC50 median values for each group. Data were analyzed by non-parametrical analysis. Asterisks indicate statistically significant difference between groups; **: *p*≤0.01, ***: *p*≤0.001, NS: not significant.

**Figure 7:**
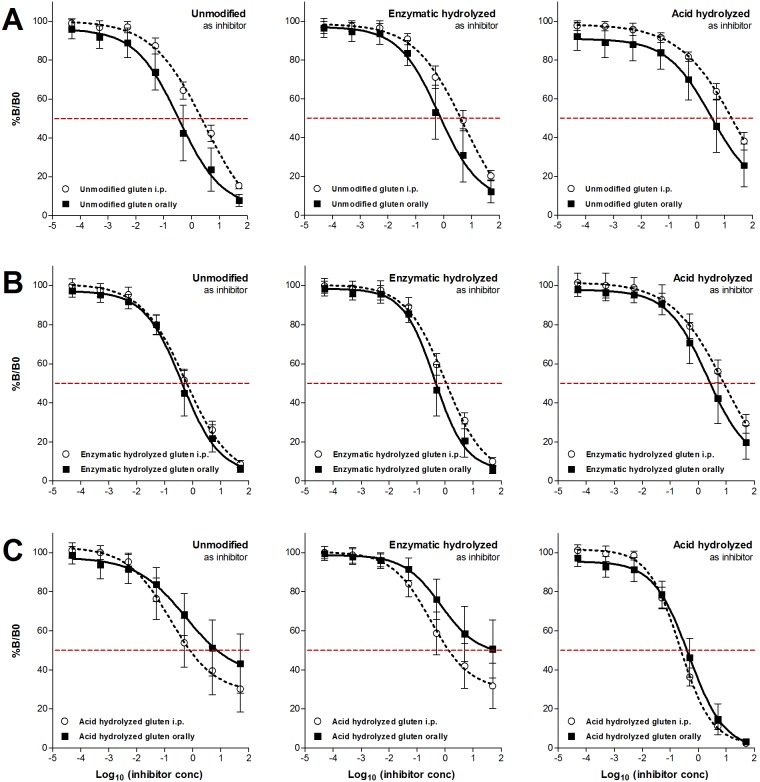
Comparison of IgG1-binding capacity in rats immunized i.p. or dosed orally with unmodified (*A*), enzymatic hydrolyzed (*B*) or acid hydrolyzed (*C*) gluten. Rats were immunized i.p. at day 0, 14 and 28 with 200 µg of gluten product per rat for i.p. studies or dosed by gavage at day 1–35 with different concentrations of gluten products for oral sensitization studies. Serum samples obtained at sacrifice were preincubated with tenfold dilutions (50 pg/mL–50 µg/mL) of unmodified, enzymatic hydrolyzed or acid hydrolyzed gluten before measurement of IgG1-binding capacity by addition to ELISA plates coated with the gluten product used for sensitization. Each graph represents mean IgG1 inhibition curves for groups of rats sensitized to the same gluten product by the i.p. (open symbols) or oral (closed symbols) route. Error bars represent ± SD.

Analysis of IgG1-binding capacity by inhibition ELISAs using sera from orally dosed rats (Figure 6) demonstrated the same pattern as found for sera from the i.p. sensitized rats (Figure 2) namely that acid hydrolysis but not enzymatic hydrolysis of gluten pro­teins generates ‘new’ epitopes. In addition, unmodified and enzymatic hydrolyzed gluten showed a tendency to produce antibodies with a stronger binding (higher avidity) after oral sensitization than after i.p. sensitization. For acid hydrolyzed gluten IgG1-binding for the two sensitization routes was identical when acid hydrolyzed gluten was used as inhibitor. With unmodified or enzymatic hydrolyzed gluten as inhibitor i.p. immunization induced antibodies with stronger binding (Figure 7).

## Discussion

Using Brown Norway rats kept on a gluten free diet for many generations and therefore naïve to gluten, we have shown that gluten and hydrolyzed gluten products can sensitize i.e. induce specific IgE using either the intraperitoneal or the oral route for dosing without the use of adjuvant. As expected i.p. immunization resulted in more responders and a higher antibody level than dosing by gavage. The i.p. route bypasses the digestive system as well as the gut-associated lymphoid tissue designed to induce tolerance to food antigens.

Using the i.p. route, immunization with the acid hydrolyzed gluten resulted in a significantly higher IgE response than both the unmodified and the enzymatic hydrolyzed gluten. When studying IgE functionality in RBL assay, sera from animals sensitized to acid hydrolyzed gluten had a lower biological activity with all three gluten products compared to sera from animals sensitized to unmodified or enzymatic hydrolyzed gluten. This result is in accordance with previous human and animal studies showing that IgE titer and biological activity may not necessarily correlate [29], [32]. In contrast, the acid hydrolyzed gluten induced an IgG1 response with a stronger binding affinity to the antigen compared to the response induced by the two other antigens. Based on the results we cannot explain this difference, but only speculate that IgE-mediated degranulation is dependent on binding of IgE to two epitopes for cross-linking with the related structural limitations. Binding measured by inhibition ELISA is possible with binding between one antibody per epitope. The results of IgG inhibition ELISAs showed that acid hydrolyzed gluten has epitopes that are not present on either unmodified or enzymatic hydrolyzed gluten. This is in line with results presented by Denery-Papini *et al*. [33] who found that IgE from patients allergic to gluten deamidated by heating and acid treatment reacted strongest with an epitope where two or three glutamines were deamidated to glutamic acid. These data supports our finding, that acid hydrolysis of gluten induces formation of new epitopes.

Gourbeyre *et al*. [6] have studied the sensitizing capacity of acid hydrolyzed gluten proteins. In this study, mice were dosed four times i.p. with 10 µg of native or deamidated gliadin together with adjuvant (Al(OH)_3_) on day 0, 10, 20 and 30. Results showed that deamidated gliadin induced a higher IgE response and higher histamine release than native gliadin. It was also shown that the native gliadin induced a more Th1 type (not allergic) response compared to deamidated gliadin. The mice used in the Gourbeyre experiments were most likely tolerant to gliadin because they were bred on a wheat-containing diet and only kept on a gluten-free diet from three weeks before experiments were initiated. Generally, rodents should be bred on an ‘allergen-free’ diet for at least three generations to obtain naïve animals with no immunological memory of the examined allergen [34]–[36]. Tolerance can be broken when immunizing i.p., but the functionality of the immune response will be influenced by the tolerance [29]. Because of the likely tolerance and in light of our finding, that acid hydrolysis of gluten proteins creates new epitopes, comparing the results between sensitization to native and deamidated gliadin based on the Gourbeyre results becomes questionable. The underlying tolerance to the native gliadin epitopes will affect the response to gliadin epitopes but not to the deamidated gliadin epitopes. Thus, the results showing that native gliadin seems to induce a Th1 type response may well be explained by the underlying tolerance and not by differences in immunogenicity *per se*. This demonstrates that it is very important to know if the animals are tolerant or not to the protein under investigation or cross-reacting proteins and results must be interpreted carefully in the light of this.

Using the more physiologically relevant oral route we showed that in contrast to the i.p. results, enzymatic hydrolyzed gluten is a better sensitizer in the high dose group than both unmodified and acid hydrolyzed gluten whereas there is no difference in sensitizing capacity between the three proteins in the low and medium group. The IgE response was not high enough to allow testing for functionality in RBL assay.

Recently, Adachi *et al*. [37] showed that mice bred and kept on a standard rodent diet were sensitized to acid hydrolyzed gluten but not to unmodified gluten after transdermal exposure. Administration of gluten together with SDS increased sensitization capacity of gluten thus indicating that an increased solubility or transdermal permeability of gluten may be important for sensitization. In Japan, the use of facial soap containing acid hydrolyzed wheat has resulted in many cases of sensitization pointing to the skin or mucosal surfaces as the likely route of sensitization [20], [21]. The most important risk factor for developing wheat allergy in persons using facial soap with acid hydrolyzed wheat was the number of soap bars used [38]. In contrast to the European experience, Japanese patients also developed allergy to wheat as WDEIA [20], [21]. In an experiment, Strid *et al*. [39] were able to break oral tolerance in mice when the allergen (peanut) was dosed on tape stripped skin. It seems that in the Japanese patients, allergic skin reactions caused by the acid hydrolyzed wheat protein have contributed to break the oral tolerance to epitopes on native wheat.

We have used commercial enzyme and commercial acid hydrolyzed gluten. Although our results on acid hydrolyzed gluten are supported by others [6], [21], [24], generalization of results between different enzyme hydrolyzed products or different acid hydrolyzed products may be done with caution. The results by Denery-Papini *et al*. [24] show the degree of deamidation to be crucial for the changed allergenic properties of (acid) hydrolyzed gluten. Our results support these findings.

In conclusion, we have shown that in rats not tolerant to gluten i.p. immunization results in statistically significant difference in the specific IgE response with acid hydrolyzed > enzymatic hydrolyzed > unmodified gluten. This result is not reflected in the biological reactivity where unmodified = nzymatic hydrolyzed > acid hydrolyzed gluten. In the more physiological relevant oral route enzymatic hydrolyzed gluten gives a higher IgE response in the high dose group. There is no difference between the proteins in the low and medium dose groups. As we do not know the dose-response relationship in sensitization it is difficult to interpret the significance of the different dose-response relations in IgE response between unmodified and acid hydrolyzed gluten (no dose response) and enzymatic hydrolyzed gluten (clear dose response).

The results also support the existence of ‘new’ epitopes formed by deamidation on acid hydrolyzed gluten compared to unmodified and enzymatic hydrolyzed gluten. This makes it possible to be tolerant to gluten (be able to eat wheat products) and develop an allergic reaction to acid hydrolyzed gluten. There are several reports describing sensitization in wheat tolerant humans to hydrolyzed gluten via the skin [7], [14], [15], [18]–[21]. It is not always very clear whether sensitization in wheat tolerant subjects was caused by acid hydrolyzed or enzyme hydrolyzed gluten because the type of hydrolysis is not always clearly specified. Our results indicate that sensitization to acid hydrolyzed gluten is possible in wheat tolerant subjects because of new epitopes. Likewise our results indicate that sensitization to enzyme hydrolyzed gluten is unlikely in wheat tolerant subjects because of the identical epitopes. As with other proteins, our results show that a sensitization route bypassing the gut is more efficient for all three gluten products tested. This difference is most striking for acid hydrolyzed gluten.

## References

[1] Belitz H-D, Grosch W, Schieberle P (2004) Food Chemistry, 3rd revised Edn, Berlin: Springer.

[2] Shils ME, Shike M, Ross AC, Caballero B, Cousins RJ (2006) Modern nutrition in health and disease, 10th Edition, 50th anniversary Edition. Philadelphia: Lippincott Williams and Wilkins, Wolters Kluwer.

[3] Wieser H (2007) Chemistry of gluten proteins. Food Microbiol 24: 115–119. 17008153 10.1016/j.fm.2006.07.004

[4] Tatham AS, Shewry PR (2008) Allergens in wheat and related cereals. Clin Exp Allergy 38: 1712–1726. 18823308 10.1111/j.1365-2222.2008.03101.x

[5] Mills ENC, Sancho AI, Rigby NM, Jenkins JA, Mackie AR (2009) Impact of food processing on the structural and allergenic properties of food allergens. Mol Nutr Food Res 53: 963–969. 19603402 10.1002/mnfr.200800236

[6] Gourbeyre P, Denery-Papini S, Larré C, Gaudin JC, Brossard C, et al (2012) Wheat gliadins modified by deamidation are more efficient than native gliadins in inducing a Th2 response in Balb/c mice experimentally sensitized to wheat allergens. Mol Nutr Food Res 56: 336–344. 22147540 10.1002/mnfr.201100353

[7] Varjonen E, Petman L, Mäkinen-Kiljunen S (2000) Immediate contact allergy from hydrolyzed wheat in a cosmetic cream. Allergy 55: 294–296. 10753023 10.1034/j.1398-9995.2000.00516.x

[8] Finley JW, Hopkins DT (1985) Digestibility and amino acid availability in cereals and oilseeds. Minnesota, St. Paul: American Association of Cereal Chemists.

[9] Mittag D, Niggemann B, Sander I, Reese I, Fiedler E-M, et al (2004) Immunoglobulin E-reactivity of wheat-allergic subjects (baker’s asthma, food allergy, wheat-dependent, exercise-induced anaphylaxis) to wheat protein fractions with different solubility and digestibility. Mol Nutr Food Res 48: 380–389. 15672478 10.1002/mnfr.200400016

[10] Kumagai H, Suda A, Sakurai H, Kumagai H, Arai S, et al (2007) Improvement of digestibility, reduction in allergenicity, and induction of oral tolerance of wheat gliadin by deamidation. Biosci Biotechnol Biochem 71: 977–985. 17420594 10.1271/bbb.60645

[11] Lauriére M, Pecquet C, Boulenc E, Bouchez-Mahiout I, Snégaroff J, et al (2007) Genetic differences in omega-gliadins involved in two different immediate food hypersensitivities to wheat. Allergy 62: 890–896. 17620066 10.1111/j.1398-9995.2007.01456.x

[12] Palosuo K (2003) Update on wheat hypersensitivity. Curr Opin Allergy Clin Immunol 3: 205–209. 12840704 10.1097/00130832-200306000-00009

[13] Leduc V, Moneret-Vautrin D-A, Guerin L, Morisset M, Kanny G (2003) Anaphylaxis to wheat isolates: immunochemical study of a case proved by means of double-blind, placebo-controlled food challenge. J Allergy Clin Immunol 111: 897–899. 12704375 10.1067/mai.2003.1345

[14] Lauriére M, Pecquet C, Bouchez-Mahiout I, Snégaroff J, Bayrou O, et al (2006) Hydrolysed wheat proteins present in cosmetics can induce immediate hypersensitivities. Contact Dermatitis 54: 283–289. 16689814 10.1111/j.0105-1873.2006.00830.x

[15] Bouchez-Mahiout I, Pecquet C, Kerre S, Snégaroff J, Raison-Peyron N, et al (2010) High molecular weight entities in industrial wheat protein hydrolysates are immunoreactive with IgE from allergic patients. J Agric Food Chem 58: 4207–4215. 20196607 10.1021/jf903973x

[16] Pelkonen AS, Mäkinen-Kiljunen S, Hilvo S, Siltanen M, Mäkelä MJ (2011) Severe allergic reaction to gluten hydrolysate without reaction to wheat. Ann Allergy Asthma Immunol 106: 343–344. 21457885 10.1016/j.anai.2011.01.003

[17] Shinoda J, Inomata N, Chinuki Y, Morita E, Ikezawa Z (2012) Case of allergy due to hydrolyzed wheat proteins in commercial boiled pork. J Dermatol 39: 724–726. 22132762 10.1111/j.1346-8138.2011.01397.x

[18] Pecquet C, Bayrou O, Vigan M, Raison N, Lauriére M (2004) Hydrolysed wheat protein: a new allergen in cosmetics and food. Contact Dermatitis 50: 182–183. 10.1111/j.0105-1873.2006.00830.x 16689814

[19] Chinuki Y, Murata S, Morita E (2011) A case of wheat-dependent exercise-induced anaphylaxis sensitized with hydrolysed wheat protein in a soap. Contact Dermatitis 65: 55–57. 21658062 10.1111/j.1600-0536.2011.01917.x

[20] Fukutomi Y, Itagaki Y, Taniguchi M, Saito A, Yasueda H, et al (2011) Rhinoconjunctival sensitization to hydrolysed wheat protein in facial soap can induce wheat-dependent excersise-induced anaphylaxis. J Allergy Clin Immunol 127: 531–533. 21094523 10.1016/j.jaci.2010.09.035

[21] Nakamura R, Nakamura R, Sakai S, Adachi R, Hachisuka A, et al (2013) Tissue transglutaminase generates deamidated epitopes on gluten, increasing reactivity with hydrolysed wheat protein-sensitized IgE. Allergy Clin Immunol 132: 1436–1438. 10.1016/j.jaci.2013.07.017 23992746

[22] Battais F, Mothes T, Moneret-Vautrin DA, Pineau F, Kanny G, et al (2005) Identification of IgE-binding epitopes on gliadins for patients with food allergy to wheat. Allergy 60: 815–821. 15876313 10.1111/j.1398-9995.2005.00795.x

[23] Pastorello EA, Farioli L, Conti A, Pravettoni V, Bonomi S, et al (2007) Wheat IgE-mediated food allergy in European patients: α-amylase inhibitors, lipid transfer proteins and low-molecular-weight glutenins. Int Arch Allergy Immunol 144: 10–22. 17496422 10.1159/000102609

[24] Denery-Papini S, Bodinier M, Pineau F, Triballeau S, Tranquet O, et al (2011) Immunoglobulin-E-binding epitopes of wheat allergens in patients with food allergy to wheat and in mice experimentally sensitized to wheat proteins. Clin Exp Allergy 41: 1478–1492. 21771117 10.1111/j.1365-2222.2011.03808.x

[25] Bohle B (2004) T lymphocytes and food allergy. Mol Nutr Food Res 48: 424–433. 15508177 10.1002/mnfr.200400003

[26] Berin MC, Sicherer S (2011) Food allergy: mechanisms and therapeutics. Curr Opin Immunol 23: 794–800. 21943957 10.1016/j.coi.2011.08.010

[27] Cianferoni A, Spergel JM (2009) Food allergy: review, classification and diagnosis. Allergol Int 58: 457–66. 19847094 10.2332/allergolint.09-RAI-0138

[28] Barkholt V, Jensen AL (1989) Amino acid analysis: determination of cysteine plus half-cysteine in proteins after hydrochloric acid hydrolysis with a disulfide compound as additive. Anal Biochem 177: 318–322. 2729552 10.1016/0003-2697(89)90059-6

[29] Kroghsbo S, Bøgh KL, Rigby NM, Mills EN, Rogers A, et al (2011) Sensitization with 7S globulins from peanut, hazelnut, soy or pea induces IgE with different biological activities which are modified by soy tolerance. Int Arch Allergy Immunol 155: 212–224. 21282960 10.1159/000321200

[30] Pilegaard K, Madsen C (2004) An oral Brown Norway rat model for food allergy: comparison of age, sex, dosing volume, and allergen preparation. Toxicology 196: 247–257. 15036751 10.1016/j.tox.2003.11.010

[31] Kroghsbo S, Madsen C, Poulsen M, Schrøder M, Kvist PH, et al (2008) Immunotoxicological studies of genetically modified rice expressing PHA-E lectin or Bt toxin in Wistar rats. Toxicology 245: 24–34. 18215453 10.1016/j.tox.2007.12.005

[32] Shreffler WG, Beyer K, Chu TH, Burks AW, Sampson HA (2004) Microarray immunoassay: association of clinical history, in vitro IgE function, and heterogeneity of allergenic peanut epitopes. J Allergy Clin Immunol 13: 776–782. 10.1016/j.jaci.2003.12.588 15100687

[33] Denery-Papini S, Bodinier M, Larré C, Brossard C, Pineau F, et al (2012) Allergy to deamidated gluten in patients tolerant to wheat: specific epitopes linked to deamidation. Allergy 67: 1023–32. 22737987 10.1111/j.1398-9995.2012.02860.x

[34] Christensen HR, Brix S, Frøkiaer H (2004) Immune response in mice to ingested soya protein: antibody production, oral tolerance and maternal transfer. Br J Nutr 91: 725–732. 15137924 10.1079/BJN20041093

[35] Brix S, Christensen HR, Barkholt V, Frøkiaer H (2005) Effect of maternal dietary cow’s milk on the immune response to beta-lactoglobulin in the offspring: a four-generation study in mice. Int Arch Allergy Immunol 136: 250–257. 15722634 10.1159/000083951

[36] De Jonge JD, Knippels LM, Ezendam J, Odink J, Penninks AH, et al (2007) The importance of dietary control in the development of a peanut allergy model in Brown Norway rats. Methods 41: 99–111. 17161306 10.1016/j.ymeth.2006.09.004

[37] Adachi R, Nakamura R, Sakai S, Fukutomi Y, Teshima R (2012) Sensitization to acid-hydrolyzed wheat protein by transdermal administration to BALB/c mice, and comparison with gluten. Allergy 67: 1392–1399. 22994386 10.1111/all.12018

[38] Fukutomi Y, Kishikawa R, Sugiyama A, Minami T, Taniguchi M, et al. (2014) Akiyama K. Risk factors for the development of wheat allergy among individuals who have used a facial soap containing hydrolysed wheat protein: case-control study. EAACI Copenhagen Abstract 1617.

[39] Strid J, Hourihane J, Kimber I, Callard R, Strobel S (2005) Epicutaneous exposure to peanut protein prevents oral tolerance and enhances allergic sensitization. Clin Exp Allergy 35: 757–766. 15969667 10.1111/j.1365-2222.2005.02260.x

